# Epitope screening and self-assembled nanovaccine molecule design of PDCoV-S protein based on immunoinformatics

**DOI:** 10.3389/fmicb.2024.1402963

**Published:** 2024-06-06

**Authors:** Yaping Chen, Xinqi Song, Wenshuang Chen, Xinyi Zhao, Li Yang, Dongyu Liu

**Affiliations:** College of Animal Science and Technology, Heilongjiang Bayi Agricultural University, Daqing, Heilongjiang, China

**Keywords:** porcine delta coronavirus, S protein, immunoinformatics, self-assembled, nanovaccine

## Abstract

Based on the whole virus or spike protein of pigs, δ coronavirus (PDCoV) as an immunogen may have unrelated antigenic epitope interference. Therefore, it is essential for screening and identifying advantageous protective antigen epitopes. In addition, immunoinformatic tools are described as an important aid in determining protective antigenic epitopes. In this study, the primary, secondary, and tertiary structures of vaccines were measured using ExPASy, PSIPRED 4.0, and trRosetta servers. Meanwhile, the molecular docking analysis and vector of the candidate nanovaccine were constructed. The immune response of the candidate vaccine was simulated and predicted using the C-ImmSim server. This experiment screened B cell epitopes with strong immunogenicity and high conservation, CTL epitopes, and Th epitopes with IFN-γ and IL-4 positive spike proteins. Ferritin is used as a self-assembled nanoparticle element for designing candidate nanovaccine. After analysis, it has been found to be soluble, stable, non-allergenic, and has a high affinity for its target receptor, TLR-3. The preliminary simulation analysis results show that the candidate nanovaccine has the ability to induce a humoral and cellular immune response. Therefore, it may provide a new theoretical basis for research on coronavirus self-assembled nanovaccines. It may be an effective candidate vaccine for controlling and preventing PDCoV.

## 1 Introduction

Porcine deltacorona (PDCo) is a highly contagious, acute, and fatal respiratory and gastrointestinal infection of pigs caused by porcine delta coronavirus (PDCoV) (Ma et al., 2015; Wang et al., 2016; Liu and Wang, 2021). PDCoV can infect pigs of all ages with typical symptoms, including vomiting, watery diarrhea, and dehydration. The mortality rate of nursing piglets after infection is relatively high, causing huge economic losses to pig breeding (Chen et al., 2015; Ma et al., 2015; Jung et al., 2016; Zhang, 2016; Liu et al., 2020). It is reported that PDCoV also has the characteristics of cross-species transmission, which can lead to the risk of zoonosis and pose a potential threat to human health (Liang et al., 2019; Boley et al., 2020). Vaccination is a better approach, providing a barrier of protection against possible virus attacks and gradually promoting virus eradication (Beverley, 2002). At present, the research focus is on a polyvalent virus vector vaccine to develop a vaccine with higher safety and a better immune effect for PDCoV. However, its production cycle and costs have increased significantly. Therefore, it is particularly important to vigorously promote the research, development, and improvement of new vaccines for the prevention and control of PDCoV.

The immune response induced by the vaccine is a complex process that includes antigen capture by antigen-presenting cells (APCs), endosome escape, epitope production, and epitope presentation, thus inducing antigen-specific adaptive immunity (Guimarães et al., 2015). Virus-infected cells are usually killed by the cytotoxic effects of CTL. CTL uses allele presentation epitopes as signals (Yap et al., 1979; Lukacher et al., 1984). In general, using the allelic presentation of helper T cell epitopes to activate B cells to produce antibodies. Inoculation of vaccines triggers autoimmune responses that result in the development of autoantibodies (Guimarães et al., 2015). Therefore, viral epitopes are ideal candidates for building vaccines that can trigger the body’s immune system to produce an immune response to the virus. Using the immunoinformatics method to predict epitopes of B cells and T cells can promote the development of antiviral vaccines. In recent years, epitope vaccines have become a new form of vaccine, especially in animal vaccines (Martínez-Archundia et al., 2022). However, an efficient delivery platform is needed due to the short target antigen and weak antigenicity of epitope vaccines. Self-assembled nanocarrier vaccines provide new ideas, such as ferritin, a cage-like protein of non-viral origin (Bhushan et al., 2014). It can self-assemble into highly ordered polymers and form multiple surfaces to display antigenic epitopes, which can greatly improve immunogenicity and vaccine efficacy. It has low heterogeneity, which provides the basis for ferritin as a nanovaccine carrier (Templeton, 2002; Li et al., 2019).

PDCoV belongs to the Coronaviridae coronavirus genus of Nidovirales. PDCoV is a no-segment and single-stranded plus-stranded RNA virus with a capsule membrane (Liu et al., 2020). The full-length genome of PDCoV is approximately 25.4 kb, encoding 15 mature non-structural proteins (nsp2-16), 4 structural proteins (spike (S) protein, envelope (E) protein, membrane (M) protein, and nucleocapsid (N) protein), and 3 accessory proteins (NS6, NS7, and NS7a) (Wang et al., 2019; Liu et al., 2020). As a kind of trimeric fusion protein, S protein plays an important role in virus binding to cell receptors, mediating virus invasion and infection (Li and Goff, 2015). The interaction between the viral S protein and the receptor largely determines the host spectrum and tissue tropism of coronaviruses. It is the main antigen that the virus induces in the body to produce neutralizing antibodies, making it one of the most promising targets for vaccine design (Ji et al., 2022). Therefore, this study aimed to screen and design self-assembled nanovaccines targeting S protein through immunoinformatics and develop potential candidate vaccines against PDCoV.

## 2 Materials and methods

**Figure 1:**
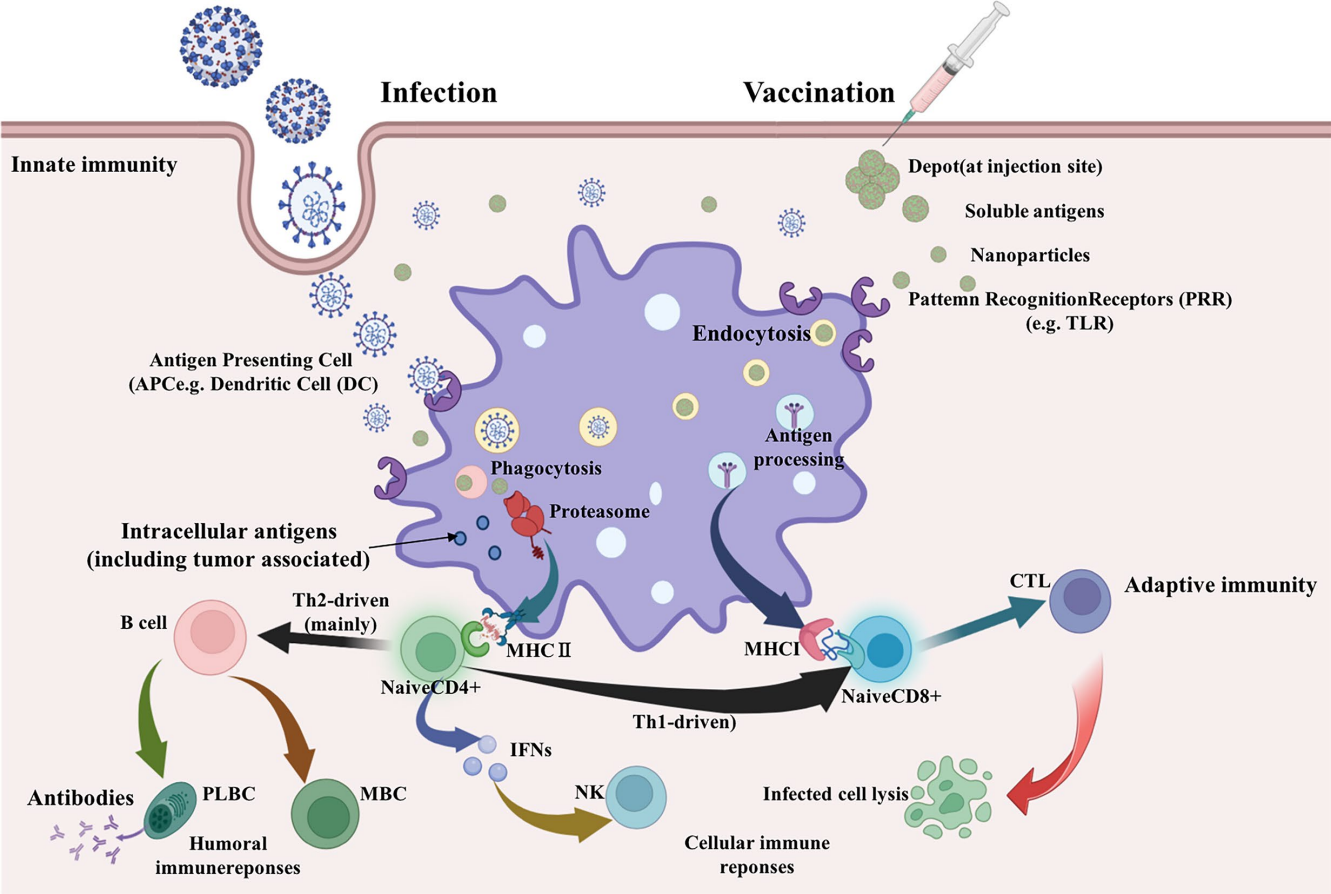
Main pathways of vaccine immune response (Biorender plot).

A schematic diagram of the main pathways of vaccine immune response based on epitopes has been summarized, as shown in Figure 1. The prediction workflow is carried out according to theory.

### 2.1 Identification of PDCoV-S protein candidate vaccine strains

The spike glycoprotein published in the database [corcine deltacoronavirus] (GenBank: AXP32216.1) can be considered a template for predicting cell epitopes. A total of 78 amino acid sequences of PDCoV-S protein with different sequences were collected in FASTA file format through the NCBI database.^1^ Multiple sequence alignment was performed using MEGA7.0 software to prepare a conservative analysis template.

### 2.2 Prediction of T and B cell epitopes

The prediction of helper T cells (/CD4+) epitopes is the inability to obtain applicable data, and the MHC haplotype anchor residue regions of human HLA alleles and pig MHC alleles are comparable. Therefore, the interaction between HTL epitopes and different MHCII class HLA alleles was predicted through the NetMHCIIpan-4.0 server^2^ (Reynisson et al., 2020). When the length of the epitope is adjusted to 15, the strongly bound epitope is finally selected. The binding potential between the epitope and MHC II is determined by EpiTOP^3^ (Dhanda et al., 2013). Subsequently, the inducing ability of IFN-γ and IL by the predicted epitope was predicted through the IFN epitope tool IFNepitope^4^ and IL4pred^5^ (Dimitrov et al., 2010). Screening for active epitopes can simultaneously activate these two cytokines.

The interaction between the cytotoxic T cells (/CD8+) epitopes and different MHC class SLA alleles was predicted by NetMHCpanEL4.1 server^6^ and NetMHCpan4.1 server^7^ (Reynisson et al., 2020). The epitope length is adjusted to 14, and the threshold of pig alleles approaches 0, ranking in the top 10% of strongly bound sequences.

The distribution of B cell epitopes in the entire sequence was predicted through the ABCpred server^8^ and PEPTIDES server^9^ (Buchan et al., 2013). Adjust the length of ABCpred table bits on the server to 16, with a threshold of 0.8. The PEPTIDES server determined the entire amino acid sequence and reported a minimum size of eight residues, with an accuracy of 75%. Further use of immunogenicity tools and toxicity assessment tools, IEDBMHC-I^10^ and ToxinPred,^11^ evaluates the immunogenicity and toxicity of all predicted epitopes (Gupta et al., 2013). The epitopes with an immunogenicity score of >0.1 and no toxicity were selected as the final prediction.

### 2.3 Screening and conservative evaluation of dominant epitopes

In order to select suitable epitopes for further analysis, the best fragment with high overlap was identified by comparing the B cell epitopes, Th epitopes, and CTL epitopes generated by the server. Then, the dominant epitopes were evaluated through the protection analysis tool on the IEDB server^12^ (Bui et al., 2007). The protection of antigenic epitopes based on the identified candidate epitopes and 78 different S protein sequences was analyzed, and the conservation of antigenic epitopes was calculated. The analysis type and sequence recognition threshold were set to “linear” and “100%,” respectively, and the same template sequence was removed. Meanwhile, PyMOL software was used to plot the dominant epitope spatial positions of the PDCoV-S protein (PDB ID: 6B7N).

### 2.4 Design and evaluation of vaccine

As mentioned above, the study identified ideal candidates for CD8 + T, CD4 + T, and B cell epitopes. The vaccine construction plan is to use AAY, GPGPG, and KK flexible linkers for CD8+/CTL, CD4+/HTL, and B cell epitopes, respectively. Using EAAK flexible linker to incorporate ferritin (GenBank: WP_000949190) into the C-terminus of the structural peptide was used to enhance the immunogenicity of the vaccine structure. The antigenicity, overexpression solubility, allergenicity, and physicochemical properties of the vaccine are determined through the server ANTIGENpro,^13^ SOLpro,^14^ AllerTop,^15^ and ExPASy,^16^ respectively (Gasteiger et al., 2003; Cheng et al., 2005; Dimitrov et al., 2014). The secondary structure of the vaccine has been validated through the SOPMA service^17^ and PSIPRED 4.0 service^18^ (Geourjon and Deléage, 1995; Buchan et al., 2013), respectively.

### 2.5 Prediction, refinement, and validation of the tertiary structure of the vaccine

A rough model of the three-level structure of vaccines was built through the trRosetta server^19^ (Yang et al., 2020). The rough model was refined to improve the quality of the structure using the GalaxyRefine server^20^ (Heo et al., 2013). To refine the model for greater stability, Ramachandran diagrams were generated to compare and evaluate the quality of the model before and after optimization using the PROCHECK server.^21^ The three-level structural model of the final candidate vaccine was validated through the ProSA web server^22^ (Lovell et al., 2003).

### 2.6 Molecular dynamics simulation analysis

Molecular dynamics (MD) simulation is an important tool in protein research and drug design, simulating vaccine docking complexes using Gromacs 2022.3 (Kutzner et al., 2015). Adding a GAFF force field to the vaccine was achieved using AmberTools22, using Gaussian 16 W for hydrogenation operations and calculating the RESP potential to optimize the molecular geometry and charge distribution. A simulation environment of 300 K and 1 Bar was selected, using the Amber99sb-ildn force field and Tip3p water model, and the total charge of the system was neutralized using Na^+^ ions. The energy minimization step adopts the steepest descent method to eliminate conformational stress. Subsequently, 100,000 steps (100 ps) of NVT and NPT equilibration were performed, followed by 50 million steps (100 ns) of free MD simulation. During the analysis process, physical quantities such as root mean square deviation (RMSD), root mean square fluctuation (RMSF), radius of gyration (Rg), solution accessible surface area (SASA), and hydrogen bonding of the composite were calculated, providing information on the structural stability, flexibility, and solvent accessibility of the composite. In addition, the built-in ‘gmx_sham’ function and ‘xpm2txt. Py’ foot of Gromacs 2022.3 were utilized to analyze the Ben Gibbs free energy morphology. The binding energy and thermodynamic stability between the complexes were evaluated using the MM/GBSA method (Zhu et al., 2023).

### 2.7 Immune simulation, codon optimization, and *in silico* cloning

The interaction of proteins is one of the most important means of studying protein structure and function. Therefore, the interaction between TLR-3 protein as receptor (PDB ID: 2A0Z) and vaccine as a ligand was completed through the pyDockWEB server^23^ (Jiménez-García et al., 2013). Amino acid residues with hydrogen bonding and hydrophobic interactions were identified using LigPLot+. The immunogenicity and immune response mode of the vaccine were completed through the C-ImmSim server^24^ (Rapin et al., 2010). All parameters remain at their default values and the immune process steps are set to 1, 25, and 50 (8 h per step). The vaccine code optimization and reverse translation were completed through the JCat server^25^ (Grote et al., 2005). Then, the restriction endonuclease sites were inserted into the corresponding expression vector system through SnapGene.

## 3 Results and discussion

### 3.1 Preliminary screening of PDCoV-S epitopes

**Figure 2:**
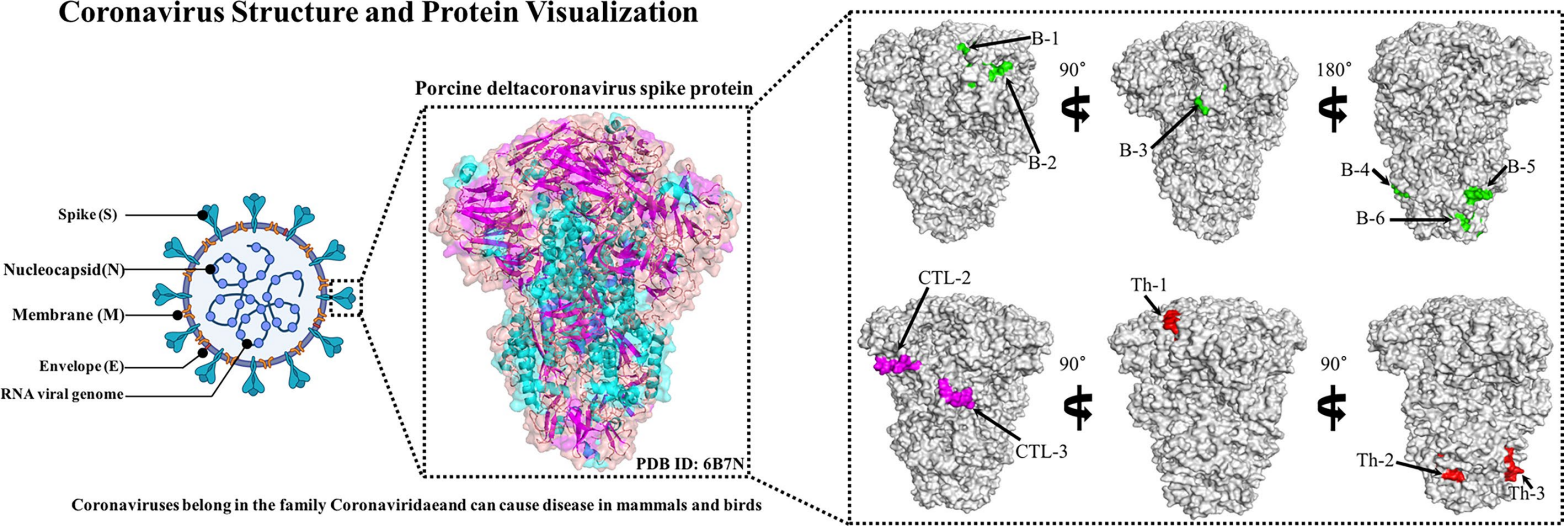
Candidate epitope spatial positions of PDCoV-S protein. The tertiary structure of S protein includes the green-labeled B cell epitope, the red-labeled Th epitope, and the purple-labeled CTL epitope.

The B cell epitopes of PDCoV-S protein were screened using the ABCpred server and PEPTIDES server, resulting in six epitopes, as shown in Table 1. The cytotoxic T cell (CTL/CD8+) epitopes were screened for the same allele using the NetMHCpanEL4.1 server and NetMHCpan4.1 server, resulting in four epitopes, as shown in Table 2. The different alleles of helper T cell (HTL/CD4+) epitopes were predicted and screened through the NetMHCIIpan-4.0 server. Then, after being identified by the EpiTOP server and analyzed using IFNepitope and IL4pred servers, it was finally determined that three epitopes can simultaneously activate IFNγ and IL, as shown in Table 3. However, the potential challenge in this study is whether there is a difference between the actual immune effect and the simulated immune effect. To avoid this situation, the advantageous epitopes we have chosen include the neutralizing epitope regions currently validated in experiments, while splicing the potential regions of the S protein, which is consistent with the current preparation of influenza virus vaccines (Jiao et al., 2023), eliminating epitopes that produce toxic side effects. In the epitope prediction strategy of this study, confirmed epitopes were analyzed for protein sequence percentage and minimum identity conservation. In order to observe the spatial position of epitopes, PyMOL software was used to display the corresponding spatial positions, as shown in Figure 2. The predicted epitopes basically cover the surface of the protein, which is conducive to stimulating the immune response. Unfortunately, the spatial positions of CTL-1 and CTL-4 epitopes have not been displayed due to incomplete protein analysis.

**Table 1:** Candidate B cell epitopes.

Name	Sequences	Length	Start position	End position	Toxin prediction	Percent of protein sequence ≦100%/%	Minimum identity/%
B1	GENYVFVCS	9	149	157	non-toxin	100.00% (78/78)	100.00%
B2	CEMKIIVTYVWNYLLRQ	17	385	407	non-toxin	96.15% (75/78)	94.12%
B3	DVCTDYTIYGVSG	13	430	442	non-toxin	91.03% (71/78)	92.31%
B4	VFGGLTAAAAIPF	13	740	752	non-toxin	97.44% (76/78)	92.31%
B5	SAGICVD	7	946	952	non-toxin	100.00% (78/78)	100.00%
B6	PQLILYQ	7	961	967	non-toxin	100.00% (78/78)	100.00%

**Table 2:** Candidate CTL cell epitopes.

Name	Sequences	Allele	Start position	Immunogenicity	Toxin prediction	Percent of protein sequence ≦100%/%	Minimum identity/%
CTL-1	DLLDLLTFPGAHRF	SLA-2*0502	21	0.5532	non-toxin	98.72% (77/78)	92.86%
CTL-2	SRANNFDVGVLPGY	SLA-3-YDY01	47	0.51746	non-toxin	41.03% (32/78)	71.43%
CTL-3	NRFTTTIVTPTFFY	SLA-1*0701 SLA-3-YTH	509	0.26549	non-toxin	80.77% (63/78)	92.86%
CTL-4	TLVDLEWLNRVETY	SLA-2-YDL02	1,080	0.24889	non-toxin	76.92% (60/78)	85.71%

**Table 3:** Candidate HTL cell epitopes.

Name	Sequences	Allele	Start position	IFN-γ inducer	IL-4 inducer	Percent of protein sequence ≦100%/%	Minimum identity/%
Th-1	QRTIVTLPKLPELEV	DRB1_1104; DRB1_1303; DRB1_0803; DRB1_0801; DRB1_1201	292	POSITIVE	Inducer	96.15% (75/78)	93.33%
Th-2	RPSIVSLYDGEVEIP	HLA-DQA10102-DQB10604; HLA-DQA10103-DQB10501; HLA-DQA10201-DQB10201; HLA-DQA10201-DQB10202; HLA-DQA10501-DQB10201	559	POSITIVE	Inducer	55.13% (43/78)	46.67%
Th-3	HAVLVPNKFTRVNAS	HLA-DPA10103-DPB10201; HLA-DPA10103-DPB12301; HLA-DPA10103-DPB10401; HLA-DPA10103-DPB10402	932	POSITIVE	Inducer	96.15% (75/78)	93.33%

### 3.2 Construction of PDCoV-S protein vaccine

**Figure 3:**
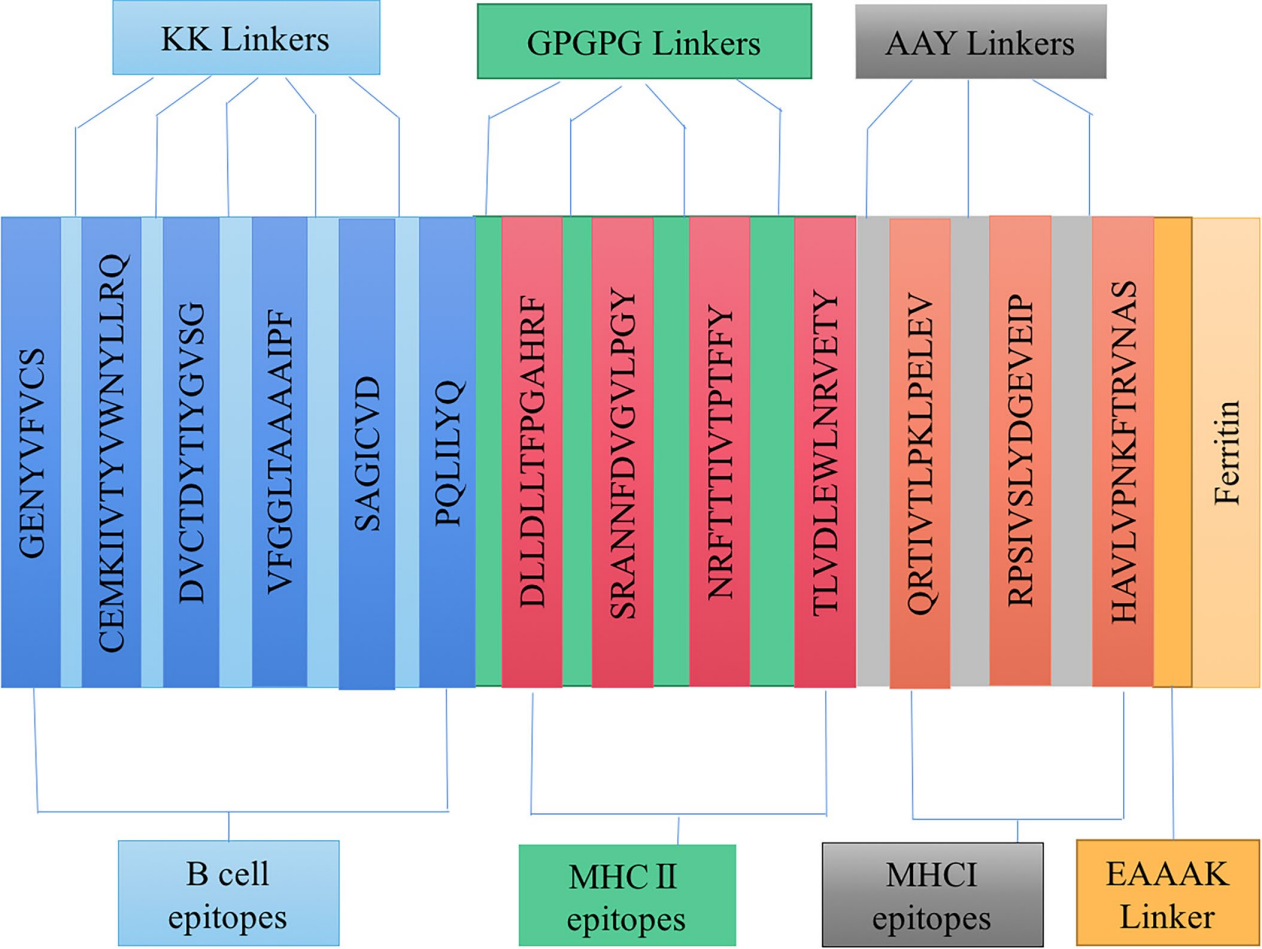
Schematic diagram of candidate vaccine construction.

Based on the epitopes obtained from the immunoinformatics analysis mentioned above, three linkers, KK, AAY, and GPGPG, were used to link the B cell, HTL, and CTL epitopes together in a certain order. A double lysine (KK) linker is used between B cell epitopes to maintain their independent immunogenicity activity and improve the immunogenicity of the candidate vaccine. Using AAY and GPGPG linkers can enhance the recognition ability of vaccine subunits, and EAAAK linkers are used to connect epitopes and ferritin. Linkers ensure good stability and folding rate; the schematic diagram of the constructed candidate vaccine is shown in Figure 3.

### 3.3 Vaccine evaluation of PDCoV-S protein

**Figure 4:**
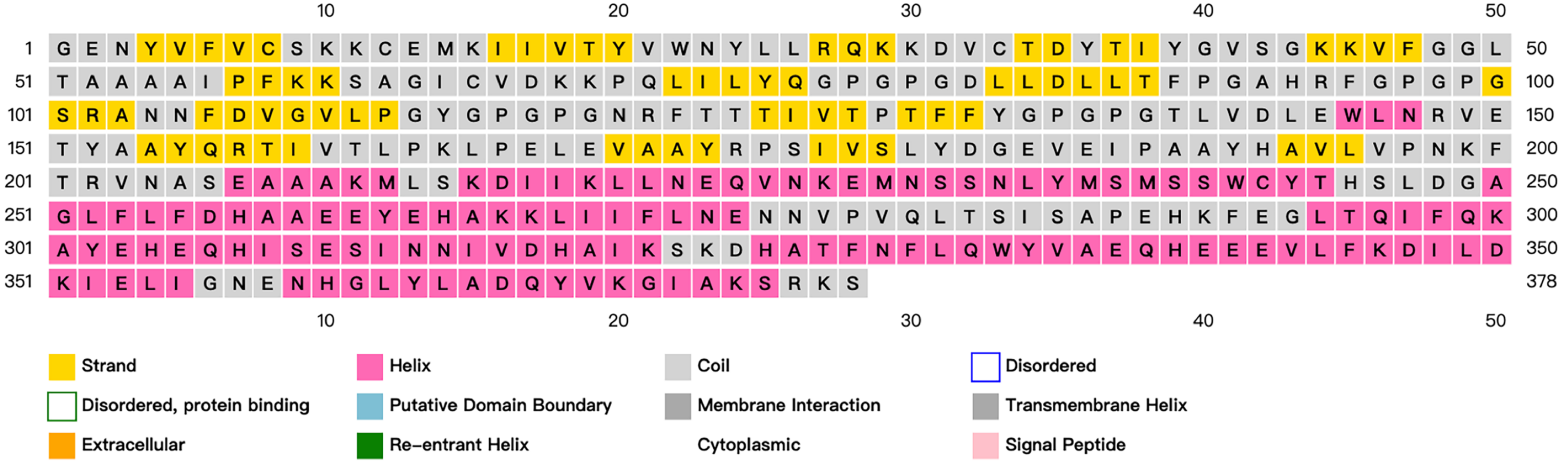
Prediction and analysis of the secondary structure of candidate vaccines. Different colors represent corresponding information about the secondary structure of a protein.

ANTIGENpro predicts an antigen immunogenicity of 0.655724, indicating that the candidate vaccine has good immunogenicity. SOLpro predicts that the overexpression solubility of candidate vaccines is 0.660427. The allergenicity of candidate vaccines is predicted by AllerTOP V2.0. The candidate vaccines are non-allergenic proteins. ExPASy calculates that the candidate vaccine has a total length of 378 amino acids, a molecular weight of 42.20924 kDa, and an isoelectric point of 6.20. The molecular formula of the candidate vaccine is C_1925_H_2959_N_493_O_555_S_10_. It has a total of 42 negatively charged residues (Asp+Glu), a total of 37 positively charged residues (Arg + Lys), and an antigen stability index of 29.63. The predicted results classify it as a stable protein. The fat index is 89.26, and the aliphatic group represents the degree of thermal stability of the protein. The prediction results of the secondary structure of candidate vaccines using PSIPRED and SOPMA server show that the candidate vaccine is mainly composed of alpha helix: 37.30%, extended strand: 25.66%, beta turn: 8.73%, and random coil: 28.31%, as shown in Figure 4.

### 3.4 Modeling, refinement, and evaluation of the tertiary structure of the candidate vaccine

**Figure 5:**
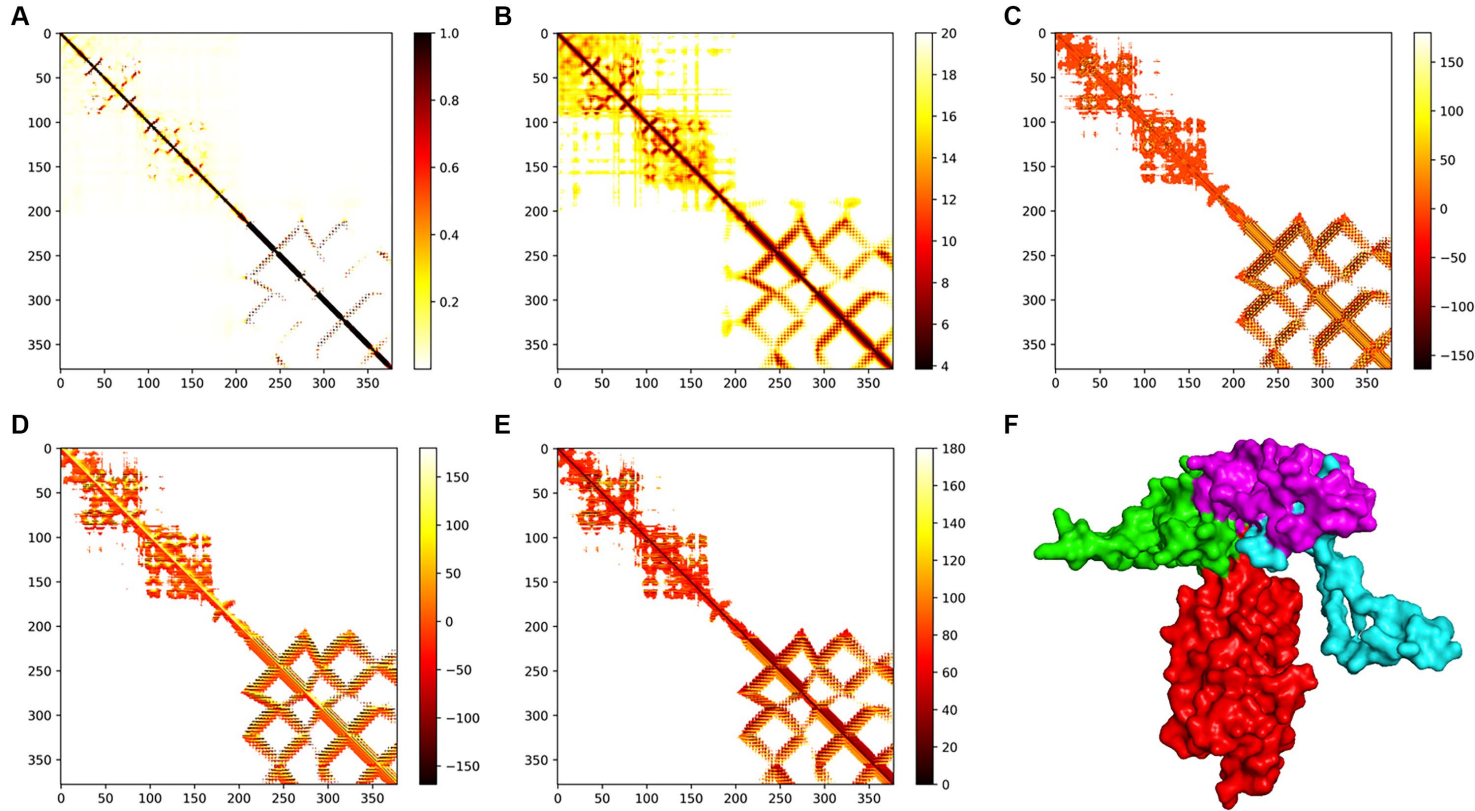
Modeling of the tertiary structure of the candidate vaccine. The parameters **(A–E)** represent the omega, phi, distance, theta, and contact parameters of the two-dimensional structure. The ferritin in the tertiary structure of the candidate vaccine is colored red, while the B cell epitope is green, the TH epitope is blue, and the CTL epitope is purple. The TM score of the structure is 0.598.

**Figure 6:**
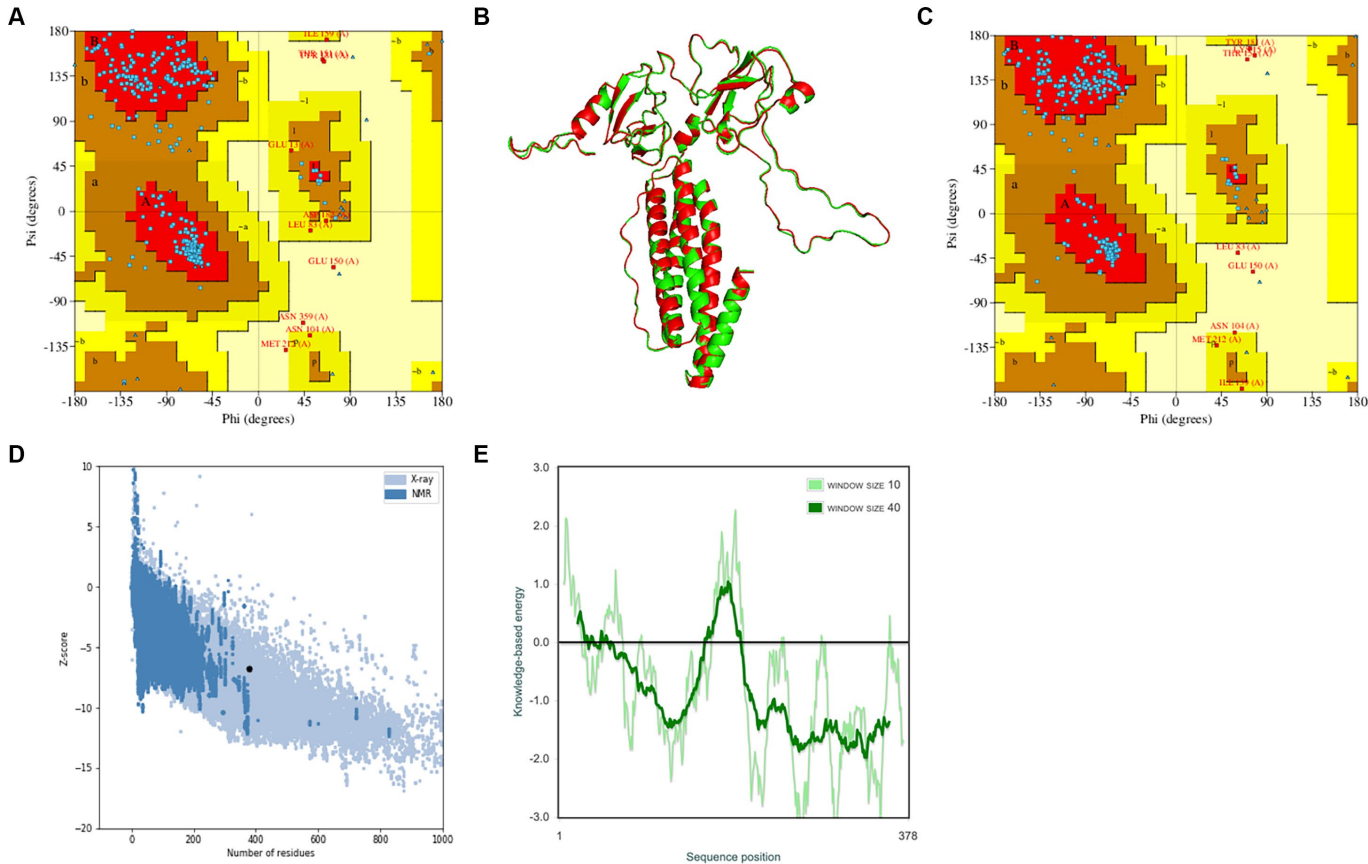
Verification of the tertiary structure of the candidate vaccine. **(A)** To verify the rough model using Ramachandran drawing; **(B)** The structure comparisons between the rough model and the refined model, and the rough model and the fine model are green and red, respectively; **(C)** To verify the refined model using Ramachandran drawing; **(D)** the Z score of the refined model ProSA SEB map is −6.73; and **(E)** local model quality assessment.

The trRosetta server models and generates a three-level structure of the candidate vaccine, as shown in Figure 5F. The server synchronously generates 2D visualization views (contact diagram, distance diagram, and direction diagram), including contact, distance, omega, theta, and phi, as shown in Figures 5A–E. The server PROCHECK was used to generate a Ramachandran graph to evaluate the quality of the coarse model. The results are shown in Figure 6A, which shows that the rough model has the most favored regions (A, B, and L) at 88.1%, additional allowed regions (a, b, l, and p) at 8.8%, generously allowed regions (~a, ~b, ~l, and ~ p) at 1.5%, and disallowed regions at 1.5%. GalaxyRefine is a server that enhances and improves a given coarse model structure. Computer software PyMOL was used to draw a cartoon comparison diagram, as shown in Figure 6B. Ramachandran plot validated the structure of the refined model, as shown in Figure 6C. The results showed that the refined model had the most favored regions (A, B, and L) at 90.0%, additional allowed regions (a, b, l, and p) at 7.6%, generously allowed regions (~a, ~b, ~l, and ~ p) 0.9%, and disallowed regions 1.5%. Compared to the coarse model, the quality of the refined model has been improved by GalaxyRefine. The server ProSA web evaluates and verifies the structure of the refined model. The structural accuracy analysis shows a z-value of −6.73, which falls within the acceptable range for natural proteins. The results are shown in Figure 6D. The local model quality’s energy score is moderate, as depicted in Figure 6E. As a result, the refined model can be used for the next analysis.

### 3.5 Docking of the candidate vaccine with TLR-3

**Figure 7:**
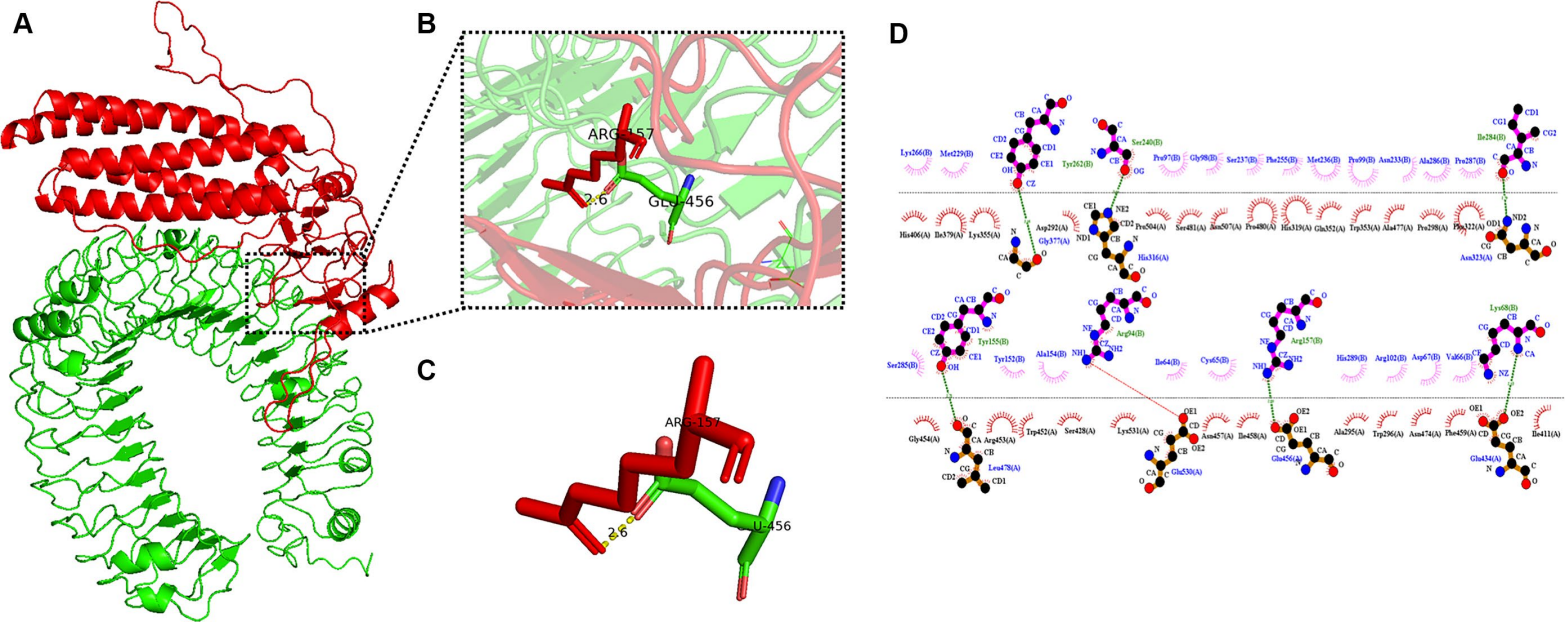
Candidates docking results of vaccine and TLR-3 molecules. **(A)** The candidate vaccine structure is shown in red and the TLR-3 structure in green; **(B)** partial zoom-in shows amino acid residues; **(C)** specific amino acid residues interacting with each other; and **(D)** schematic diagram of the interaction between protein chains.

The pyDockWEB server enables protein–protein docking between candidate vaccines as ligands and TLR-3 as receptors. A total of 500 composite models were generated for docking and model 1,313 ranked first among all prediction models. Specific values associated with this model include electrostatics: −23.424; desolvation: −23.182; Vdw: 12.950; total: −45.312. The docking results are shown in Figure 7A. The prominent amino acid residues were locally amplified, as shown in Figures 7B,C. The stable binding can be obtained under optimal conditions. LigPLot+ identified the candidate vaccine as ligands and TLR-3 as receptors, as shown in Figure 7D. The amino acid residues with hydrogen bonds were Tyr262, Ser240, Ile284, Tyr155, Arg94, Arg157, and Lys68, and the amino acid residues with hydrophobic interactions were Lys266, Met229, Fro97, Gl98, Ser237, Phe255, Asn233, Ala286, Fro287, Met236, Pro99, Ser285, Tyr52, Ala154, Ile64, Cys65, His289, Asp67, Val66, and Ar. g102.

### 3.6 *In silico* stimulation of immune responses

**Figure 8:**
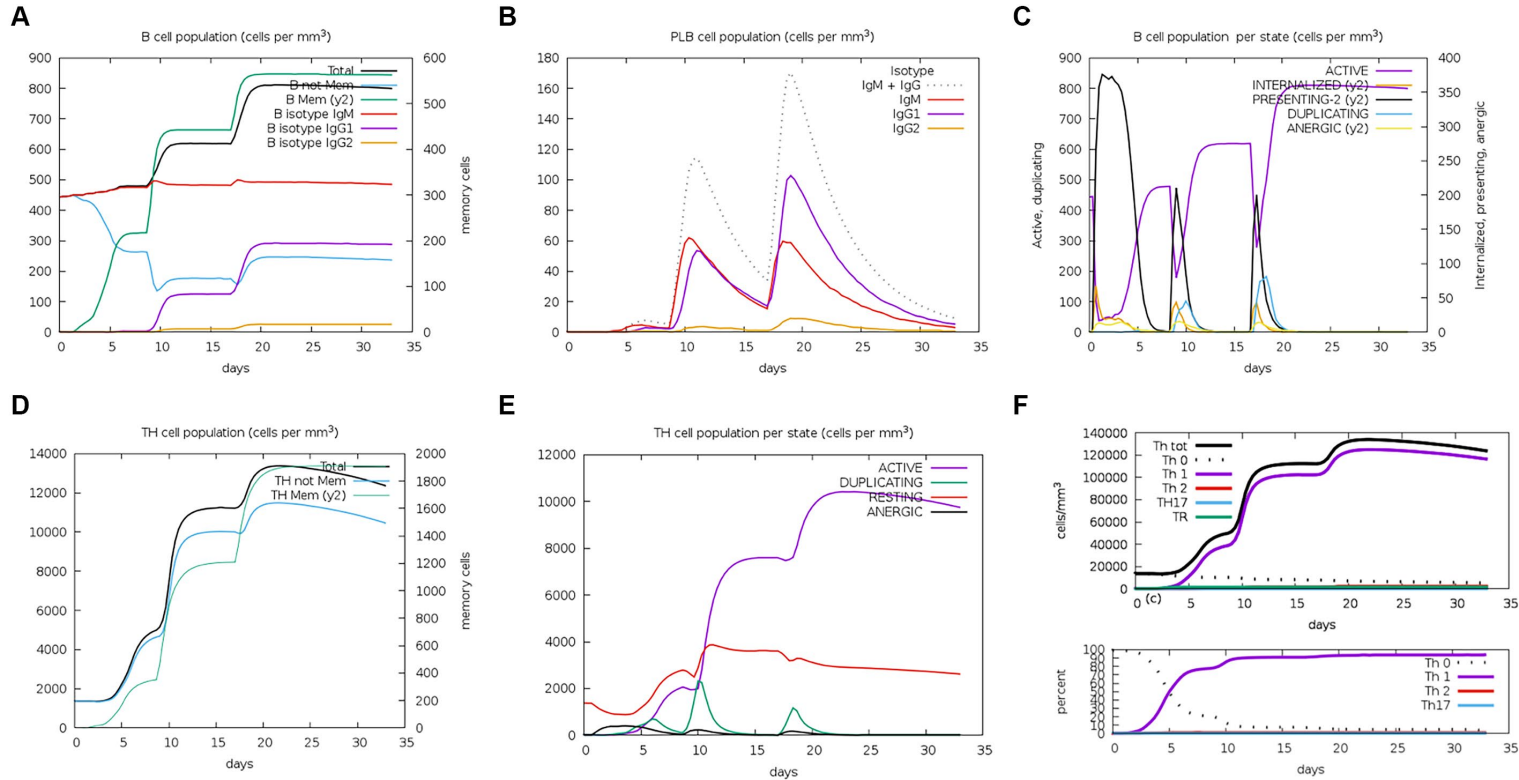
Changes in the number of immune B cells and Th in simulated immune stimulation. **(A)** The total counts of B-lymphocyte, memory cells, and subtypes IgM, IgG1, and IgG2; **(B)** the plasma B-lymphocyte count broken down by isotype (IgM, IgG1, and IgG2); **(C)** B-lymphocyte populations per entity status; and **(D)** the counts of CD4 helper T lymphocytes. The figure shows total and memory counts; **(E)** CD4 helper T lymphocyte counts per entity status; and **(F)** the counts of CD4 T-regulatory lymphocytes.

**Figure 9:**
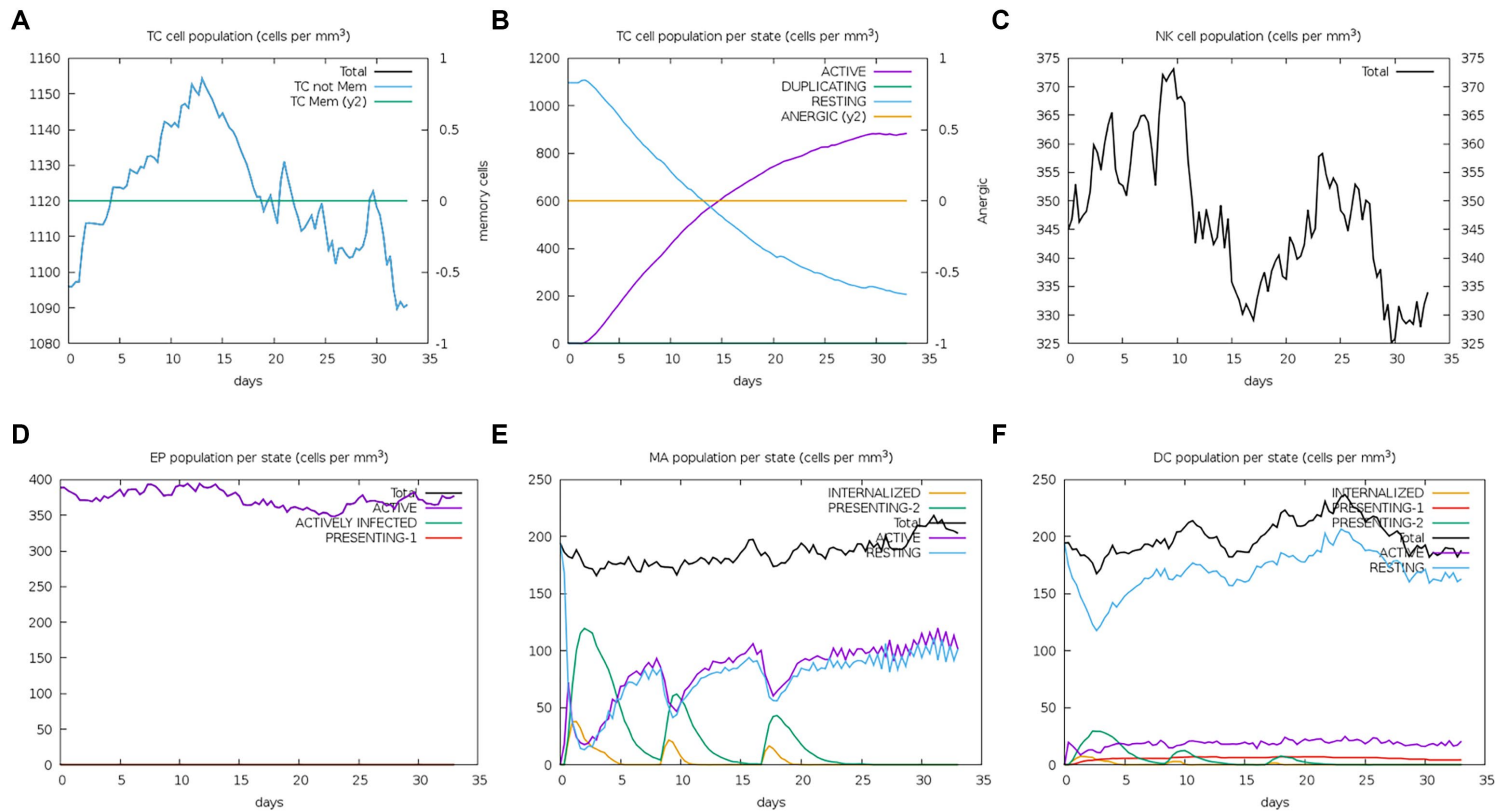
Number changes of immune CTL, NK, MA, DC, and EP cells after simulated immune stimulation. **(A)** The counts of CD8 T cytotoxic lymphocytes. Total and memory are shown; **(B)** the counts of CD8 T cytotoxic lymphocytes per entity state; **(C)** the counts of natural killer cells; **(D)** the counts of internalized and presented macrophage; **(E)** dendritic cells can present antigenic peptides on MHC class I and II molecules; **(F)** the total counts of epithelial cells can be disaggregated into active, viral infection, and presentation on MHC class I molecules.

**Figure 10:**
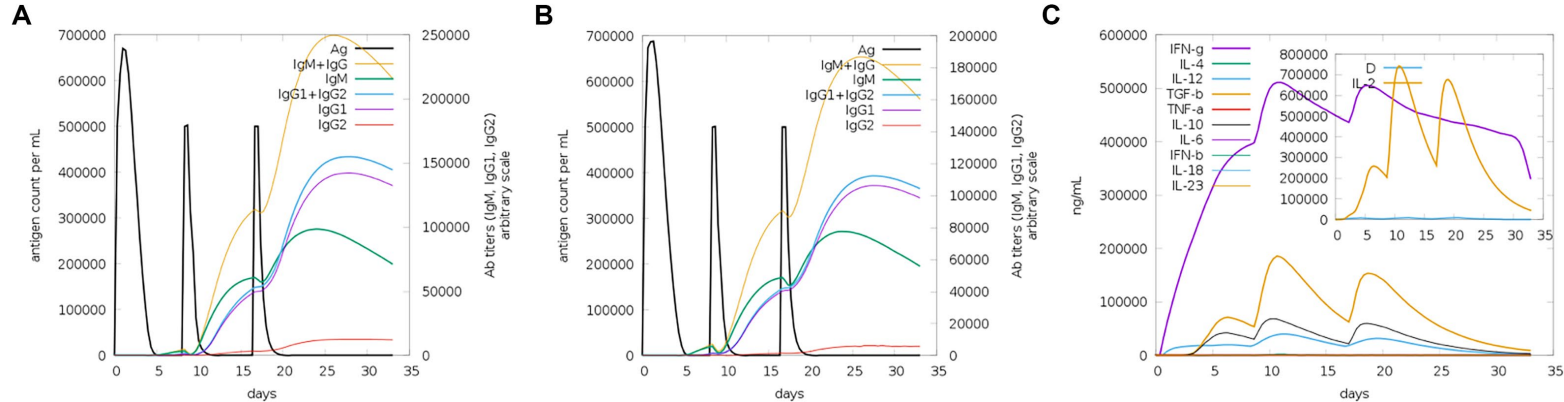
Trends of immunoglobulins and immune complexes and concentration trends of cytokines and interleukins. **(A)** Antigens and immunoglobulins. Antibodies were subdivided by isotype (containing ferritin); **(B)** antigen and immunoglobulin. Antibodies were subdivided by isotype (without ferritin); and **(C)** the concentrations of cytokines and interleukins.

The server C-ImmSim can quickly evaluate the immunogenicity of candidate vaccines through computer simulation. The results showed that the candidate vaccine could effectively stimulate the immune response of B cells, NK cells, macrophages, CD4+ T cells (Th1 and Th2), and CD8+ T cells after three stimulations. In addition, active macrophages maintained a good level in the three immune stimulations. The total number of TH cells increased significantly in the immune stimulation and reached a peak in the third injection. The population of active TH cells per state reached its highest at the third injection, and the population of non-memory TC cells peaked at 1150 cells/mm^3^. When the number of active TC cells reached a peak, the population of stationary TC cells per state was the lowest. B cells mainly produce humoral immunity in the body, and the results show that the candidate vaccine can activate B cells. The candidate vaccine induces high levels of IgM and IgG antibodies, among which IgM + IgG titer reaches 700,000, as shown in Figures 8–10. According to the simulation results of C-ImmSim, the candidate vaccine conforms to the law of inducing immune response and has good immunogenicity. It can effectively activate humoral and cellular immunities. Based on this, this study predicts and screens B cell, HTL, and CTL epitopes and concatenates them with ferritin, utilizing immunoinformatics to achieve repeated and orderly presentation of the surface of multi-faceted nanoparticles. Thus, it may stimulate immunity and translate into broader protection. The existing research suggests that the self-assembling ferritin nanoparticles coupled with linear sequences from canine distemper virus haemagglutinin protein elicit strong immune responses (Wang et al., 2022). This is consistent with the immune simulation results of this article; it was found that the immunoglobulin significantly increased through the analysis of the C-ImmSim immune stimulation test. In addition, the levels of active T cell toxicity and T helper lymphocytes significantly upgrade to enhance the immune responses. IFN-γ also remained at peak levels during the injection. This indicates that the candidate vaccine has the ability to generate immune responses and can induce the production of CD8 + T cells and CD4 + T cells, causing cellular immune responses and exhibiting good antiviral properties.

### 3.7 Molecular dynamics simulation

#### 3.7.1 Root mean square deviation, root mean square fluctuation, radius of gyration, hydrogen bonds analyses, solution accessible surface area, and Gibb’s free energy analysis

**Figure 11:**
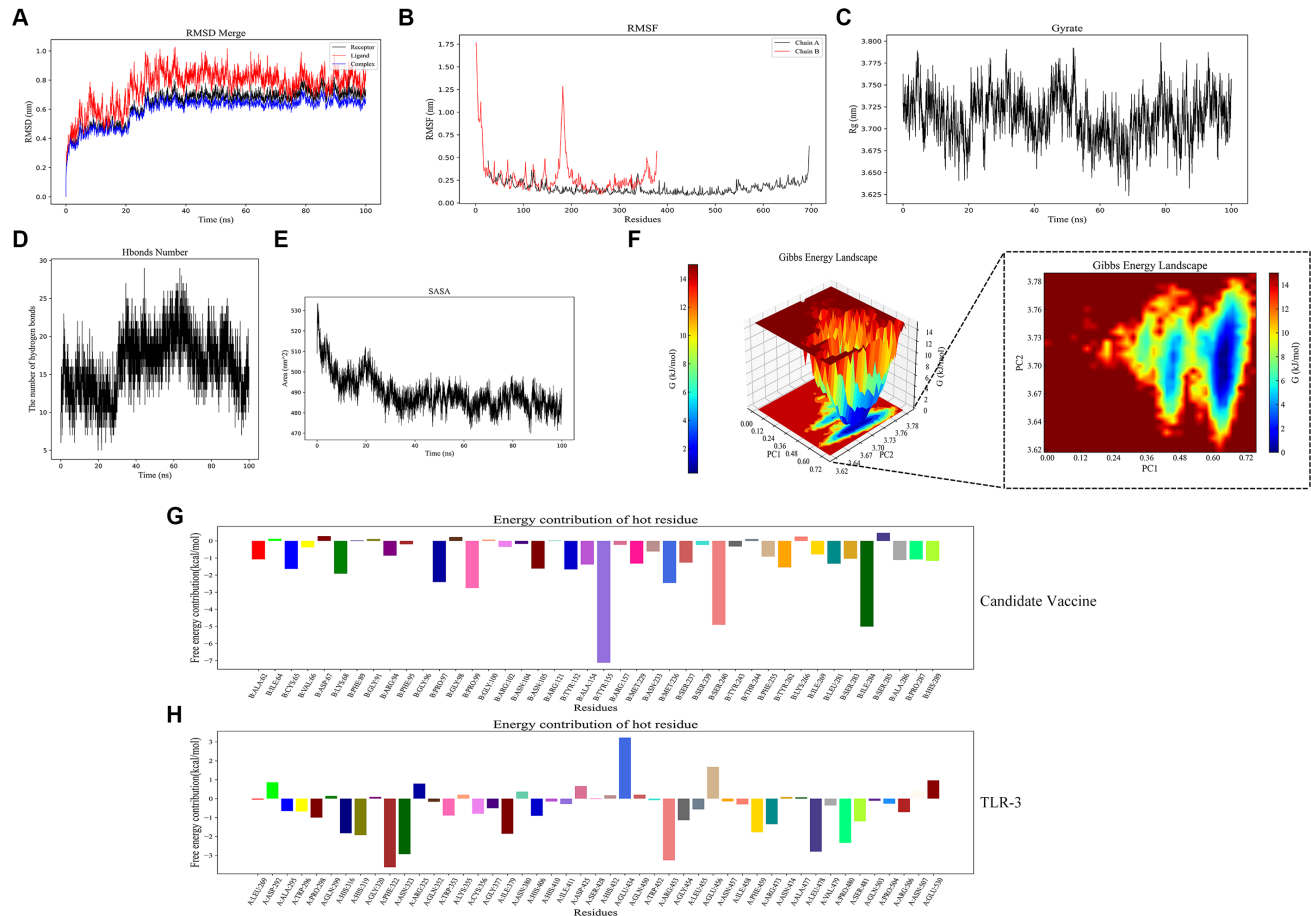
Molecular dynamics simulation. **(A)** RMSD of complexes; **(B)** RMSF of complexes, chain A represents TLR-3 and chain B represents the candidate vaccine; **(C)** Rg of complexes; **(D)** Hydrogen bonds in complexes; **(E)** SASA of complexes; **(F)** Gibb’s free energy landscape; **(G)** the free energy contribution of candidate vaccine; and **(H)** the free energy contribution of TLR-3.

RMSD and RMSF are important indicators for evaluating the stability of protein–ligand complexes. The RMSD curve shows that the composite tends to stabilize during the simulation process, indicating its structural stability. Specifically, the RMSD value of the composite remains between 0.6 and 0.8 nanometers after 25 nanoseconds (Figure 11A). The RMSF curve reflects the dynamic behavior of amino acid residues. Chain B exhibits high fluctuations in certain regions, with an RMSF value of 1.0 nanometers, indicating high flexibility. Chain A has a lower RMSF value, indicating high stability (Figure 11B). The Rg analysis further demonstrated the spatial conformation of the composite and the Rg value remained approximately 3.725 nanometers within 30–100 s, indicating a compact and stable structure (Figure 11C). Hydrogen bonding analysis revealed the dynamic changes in protein–protein interactions, with the number of hydrogen bonds fluctuating during the simulation process, reaching 20 to 25 within 20 to 60 nanoseconds, demonstrating strong interactions (Figure 11D). The SASA analysis showed a decrease from 530 square nanometers to 490 square nanometers in the first 30 nanoseconds, followed by stability, reflecting the tight binding and stability of the composite (Figure 11E). These analyses provide important references for understanding protein–ligand interactions. In Gibbs free energy analysis, the principal component analysis (PCA) of the RMSD and Rg values of the docking complex yields PC1 and PC2, which were used as the coordinate axes. Combined with Gibbs relative free energy as the Z-axis, a three-dimensional free energy landscape map was drawn (Figure 11F). When docking with TLR-3, the corresponding PC1 value fell within the range of 0.0–0.72, while the PC2 value remained stable in the range of 3.62–3.78. These values, combined with the RMSD curve of the complex, further confirm the excellent stability of the small molecule protein receptor complex.

#### 3.7.2 MM/GBSA calculation

The binding free energy and amino acid residue free energy contribution of candidate vaccines to TLR-3 were calculated by MM/GBSA. Free energy is a key thermodynamic quantity in computational biology and the calculation of free energy includes gas-phase energy (ΔGgas) and solvation-free energy (ΔGsolvation). The gas-phase energy includes van der Waals forces (ΔVDWAALS) and Coulombic forces (ΔEelec), and the solvation-free energy is obtained through an implicit solvent model. Combining the sum of free energy (ΔTotal) reflects the affinity of intermolecular interactions. The MM/GBSA calculation results of the TLR-3–amentoflavone candidate vaccine complex are shown in Table 4. The binding-free energy of this complex is −45.69 ± 41.52 kcal/mol, indicating a strong affinity between the candidate vaccine and TLR-3. The free energy decomposition of amino acids is a crucial step that helps us to gain a deeper understanding of the interactions between docking complexes. The free energy of a large number of amino acid residues is less than 0, which is beneficial for the interaction between candidate vaccines and TLR-3 (Figures 11G,H).

**Table 4:** MM/GBSA calculations of complexes.

Contribution components	TLR-3–Amentoflavone complex (kcal/mol)
ΔVDWAALS	−163.79 ± 7.86
ΔEelec	−332.47 ± 32.44
ΔEGB	471.12 ± 24.64
ΔEsurf	−20.55 ± 1.50
ΔGgas	−496.25 ± 33.38
ΔGsolvation	450.57 ± 24.69
ΔTotal	−45.69 ± 41.52

### 3.8 Codon optimization and *in silico* cloning

**Figure 12:**
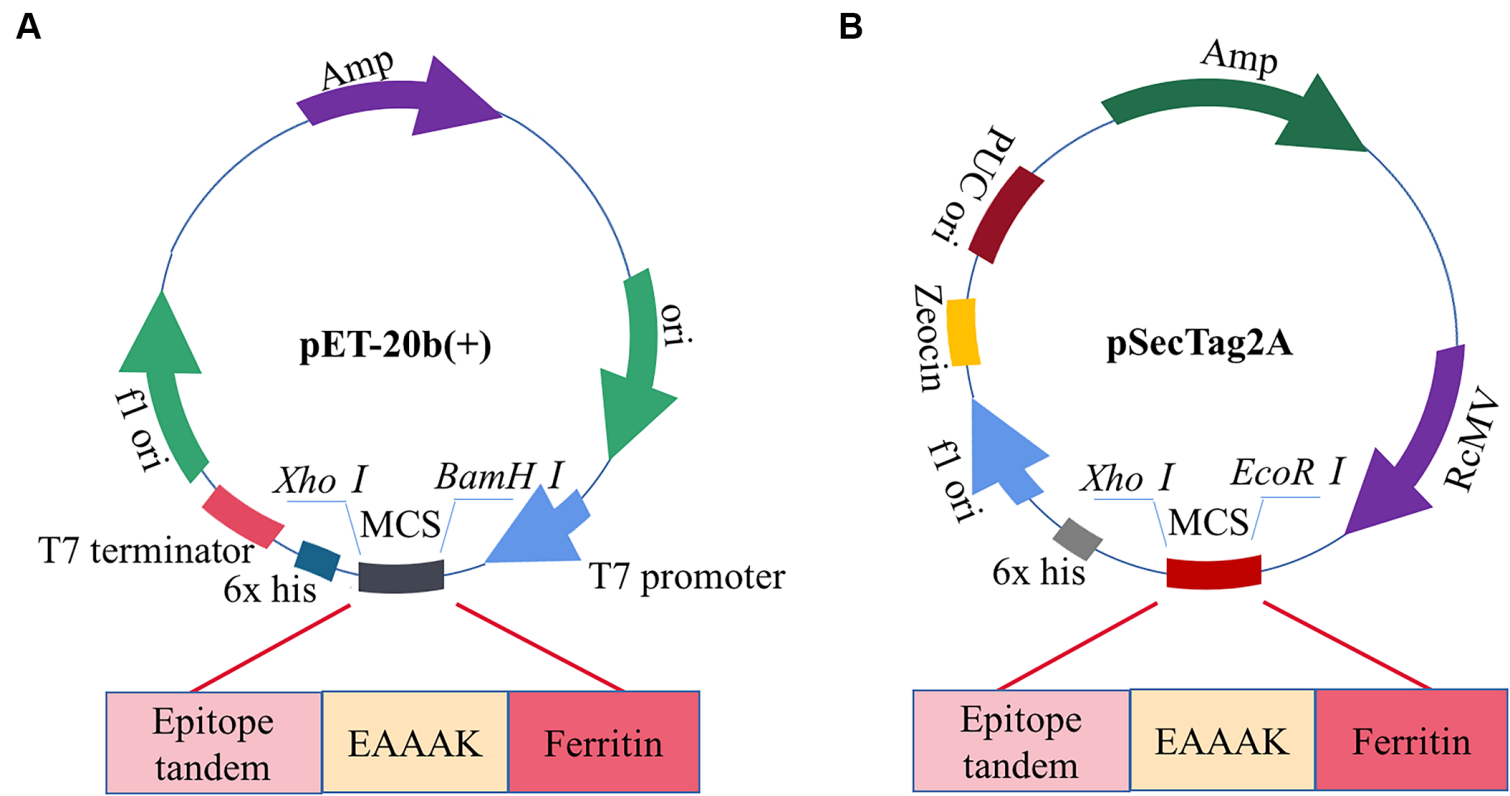
Construction of candidate vaccine expression system. **(A)** The candidate vaccine is inserted into the pET-20b (+) between *BamH I* and *Xho I* sites. **(B)** The candidate vaccine is inserted into pSecTag2A between the *EcoRI* and *XhoI* sites.

The pET-20b(+) prokaryotic expression vector was selected by computer simulation cloning. The amino acid sequence of the candidate vaccine was reverse-translated using the JCat server to obtain the expression gene sequence of the candidate vaccine, which could be better expressed by codon optimization. The improved CAI value of the optimized codon sequence was 0.97, and the GC content was 49.47%, which was in line with the optimal range of 30–70% protein expression. Therefore, the candidate vaccine prokaryotic expression plasmid was constructed, as shown in Figure 12A. We simultaneously performed eukaryotic expression of the candidate vaccine sequence and constructed eukaryotic expression plasmid, as shown in Figure 12B. It has excellent biological activity compared to prokaryotic expression. Although computer immune simulations cannot specifically present the assembly of nanoparticles, a single ferritin in tandem with multiple epitopes has already generated strong enough humoral and cellular immunity. Therefore, we guess that the actual immunity situation will be higher than we expected. Finally, the candidate vaccine was cloned using SnapGene into a prokaryotic expression vector and eukaryotic expression vector, which showed a high level of protein expression. However, in this study, the biggest challenge lies in whether vaccine molecules can be efficiently self-assembled. To avoid this situation, we have preset two commonly used expression systems (not ruling out the use of insect expression and yeast expression systems in the future). Whether the candidate nanovaccines constructed in this study can stimulate B and T cells remains to be experimentally verified. It is necessary to conduct *in vitro* and *in vivo* experiments on candidate vaccines to provide a platform for future research on the specific immune response of PDCoV nanoparticle vaccines.

## 4 Conclusion

This study successfully predicted and screened the dominant epitopes of the PDCoV-S protein using immunoinformatics and constructed a candidate nanoepitope vaccine composed of the CTL epitope, Th epitope, and linear B cell epitope that could trigger a strong immune response. The candidate vaccine, including comprehensive secondary structure analysis, tertiary structure modeling and optimization, molecular docking, codon optimization, reverse translation, and computer cloning, was evaluated. The constructed candidate nanovaccine through immunoinformatics may provide a theoretical basis against PDCoV.

## Data availability statement

The original contributions presented in the study are included in the article/supplementary material, further inquiries can be directed to the corresponding author.

## Author contributions

YC: Writing – original draft. XS: Formal analysis, Writing – original draft. WC: Methodology, Writing – original draft. XZ: Writing – review & editing. LY: Writing – review & editing. DL: Funding acquisition, Writing – original draft.

## Footnotes

^1^ https://www.ncbi.nlm.nih.gov/protocol

^2^ https://services.healthtech.dtu.dk/service.php?NetMHCIIpan-4.0

^3^ http://www.ddg-pharmfac.net/epitop/

^4^ https://webs.iiitd.edu.in/raghava/ifnepitope/index.php

^5^ http://crdd.osdd.net/raghava/il4pred/

^6^ http://tools.iedb.org/mhci/

^7^ https://services.healthtech.dtu.dk/service.php?NetMHCpan-4.1

^8^ https://webs.iiitd.edu.in/raghava/abcpred/ABC_submission.html

^9^ http://imed.med.ucm.es/Tools/antigenic.html

^10^ http://tools.iedb.org/immunogenicity/

^11^ https://webs.iiitd.edu.in/raghava/toxinpred/design.php

^12^ http://tools.iedb.org/conservancy/

^13^ http://scratch.proteomics.ics.uci.edu/

^14^ http://scratch.proteomics.ics.uci.edu/index.html

^15^ http://www.ddg-pharmfac.net/AllerTOP/

^16^ https://web.expasy.org/protparam/

^17^ http://npsa-prabi.ibcp.fr/cgi-bin/npsa_automat.pl?page=npsa_sopma.html

^18^ http://bioinf.cs.ucl.ac.uk/psipred

^19^ https://yanglab.nankai.edu.cn/trRosetta/

^20^http://galaxy.seoklab.org/cgi-bin/submit.cgi?

^21^ https://saves.mbi.ucla.edu/

^22^ https://prosa.services.came.sbg.ac.at/prosa.php

^23^ https://life.bsc.es/pid/pydockweb

^24^ https://150.146.2.1/C-IMMSIM/index.php

^25^ http://www.jcat.de/
